# Decreased brain network global efficiency after attachment memories retrieval in individuals with unresolved/disorganized attachment-related state of mind

**DOI:** 10.1038/s41598-022-08685-0

**Published:** 2022-03-18

**Authors:** Chiara Massullo, Claudio Imperatori, Fabrizio De Vico Fallani, Rita B. Ardito, Mauro Adenzato, Luigia Palmiero, Giuseppe Alessio Carbone, Benedetto Farina

**Affiliations:** 1 Department of Education, Roma Tre University, Rome, Italy; 2 Cognitive and Clinical Psychology Laboratory, Department of Human Sciences, European University of Rome, Rome, Italy; 3 Inria, Paris, France; 4 Department of Neuroscience “Rita Levi Montalcini”, University of Turin, Via Cherasco, 15, 10126 Turin, Italy; 5 Department of Psychology, University of Turin, Turin, Italy

**Keywords:** Psychology, Cognitive neuroscience, Social neuroscience

## Abstract

The main aim of the study was to examine how brain network metrics change after retrieval of attachment memories in individuals with unresolved/disorganized (U/D) attachment-related state of mind and those with organized/resolved (O/R) state of mind. We focused on three main network metrics associated with integration and segregation: global (E_glob_) efficiency for the first function, local (E_loc_) efficiency and modularity for the second. We also examined assortativity and centrality metrics. Electroencephalography (EEG) recordings were performed before and after the Adult Attachment Interview (AAI) in a sample of 50 individuals previously assessed for parenting quality. Functional connectivity matrices were constructed by means of the exact Low-Resolution Electromagnetic Tomography (eLORETA) software and then imported into MATLAB to compute brain network metrics. Compared to individuals with O/R attachment-related state of mind, those with U/D show a significant decrease in beta E_glob_ after AAI. No statistically significant difference among groups emerged in E_loc_ and modularity metrics after AAI, neither in assortativity nor in betweenness centrality. These results may help to better understand the neurophysiological patterns underlying the disintegrative effects of retrieving traumatic attachment memories in individuals with disorganized state of mind in relation to attachment.

## Introduction

Attachment trauma is of paramount importance from a clinical perspective because, along with other forms of childhood maltreatment, it is considered the most significant predictor of poor mental health across the lifespan and is also associated with clinical severity and treatment resistance, particularly because of its disorganizing and dysregulating effects on high level mental functioning^1–3^.

According to attachment theory^4^, early and repeated interactions with caregivers are encoded in memory and form internal working models (IWMs) of the self and attachment figures. IWMs are cognitive structures in which memories of past interactions with caregivers are stored and orient expectations towards their responsiveness to requests for care and comfort^4^. Although IWMs may change as a result of later attachment experiences, early IWMs appear to remain stable over time and modulate interactions with attachment figures throughout life^5,6^, providing the basis for individual differences in adult attachment strategies and states of mind.

Individuals who developed organized IWMs of attachment show, both in childhood and in adulthood, goal oriented and coherent strategies to request care and comfort^4,7,8^ and coherent states of mind when recalling attachment memories in adulthood^9^. Conversely, individuals showing an unresolved/disorganized (U/D) state of mind exhibit IWMs dominated by fearful, incoherent and contradictory expectations towards attachment figures, simultaneous or sequential contradictory behavioral strategies, poor coherence of narratives such as disoriented and/or disorganized memories of trauma or loss^9^. When activated in experimental or clinical settings, U/D IWMs usually lead to disorganized states of mind characterized by transient impairments in metacognitive monitoring, incoherence of memory, thought, and discourse concerning attachment, and a general loss of executive control over high-level mental regulation^9–11^. Furthermore, individuals with unresolved attachment-related state of mind show temporary emotion regulation breakdown (e.g., behavioral and attentional strategies lapses) when facing attachment experiences memories^12^.

U/D attachment shows a high prevalence in clinical samples in various psychiatric disorders^13^, suggesting that it could be considered a trans-diagnostic risk factor for psychopathology^14^. U/D individuals also frequently reported early childhood trauma such as repeated interactions with an overtly maltreating parent or with a neglecting, frightening, or withdrawing caregiver in early childhood^15–17^. For all these reasons, the term attachment trauma has been proposed to describe the most severe and enduring forms of U/D attachment^11,18^.

In recent decades, brain imaging techniques have been used repeatedly to elucidate mechanisms related to the pathophysiology of U/D. In particular, some studies provided preliminary evidence for the hypothesis of dysregulatory and disintegrative effects of U/D attachment on both autonomic arousal regulation and brain connectivity^19–22^, which is thought to form the neurophysiological basis of emotion regulation, risk detection, cognitive abilities, reward system, self-consciousness, social cognition, and autobiographical memory^23^.

An important advance in neuroscience research that represents a significant new approach to studying the functional and structural organization of brain connectivity is network analysis^24^. This method is based on graph theory and provides valuable information about the balance between segregation and integration processes that define brain function through a wide range of metrics^24–26^.

In the field of brain network analysis, one measure of parallel information transfer in the brain functional network is efficiency, which can be computed as global (E_glob_) and local (E_loc_) efficiency. These efficiency metrics offer several advantages for evaluating the properties of functional brain networks from both conceptual and technical perspectives^27^. Particularly, while E_glob_ is considered to assess long range information transfer referring to integration capacity, E_loc_ is a measure of short range (i.e., local) information exchanging^28^ referring to segregation capacity. Another metric that evaluates such specific capacity is modularity, which is considered an advanced measure of segregation quantifying the extent to which the whole network can be divided into non-overlapping and defined groups of nodes^29–32^.

By examining changes in the balance between segregation and integration of information^32–35^ and integrative/disintegrative processes^32,36^, these metrics may be particularly useful for understanding the pathophysiology of U/D attachment, which is thought to be characterized by dis-integration of high-level mental functioning^37–40^.

To our knowledge no studies have investigated the functional connectivity patterns associated with U/D attachment using network analysis. Therefore, the main aim of the current study was to investigate how network metrics in electroencephalography (EEG) frequency bands change after exposure to the Adult Attachment Interview (AAI) in individuals with a U/D state of mind compared to those with organized/resolved (O/R) one. Indeed, a highly demanding task such as AAI, which involves highly integrated cognitive and affective processes (e.g., autobiographical memory, metacognitive monitoring, emotional regulation)^41^, requires integrative mental activity that can be observed in large-scale cortical network^20^. For the above reasons, in this study we have focused mainly on network metrics that reflect functional integration (i.e., global efficiency) and functional segregation (i.e., local efficiency and modularity). In accordance with the view that the U/D state of mind is characterized by dis-integration of high-level mental functioning^37–40^, we hypothesized that retrieval of attachment memories in individuals with the U/D state of mind might alter network efficiency (both global and local) as well as modularity in the frequency bands (i.e., beta and gamma) involved in higher-order integrative mental functions^42–47^. Considering that network analysis offers many other measures for studying cerebral function and that “*a relatively untapped area of research is that of neurophysiological correlates of unresolved attachment pattern in adults*”^48^, we exploratively investigated other network metrics. Specifically, we computed assortativity^49^ and betweenness centrality (BC)^50^ which are, respectively, measures of resilience (i.e., the vulnerability of a network) and centrality (i.e., the relevance of nodes in a given network)^32^.

## Materials and methods

### Participants

The enrolment procedure lasted from October 2017 to May 2018 and was performed by posting advertisements around the university campus. Participants aged between 18 and 30 years were included in the study. Exclusion criteria were: (i) left-handedness, (ii) history of neurological and/or psychiatric disorders, (iii) head trauma, (iv) having taken Central Nervous System active drugs in the two weeks prior to experiment participation. Socio-demographic data, and inclusion/exclusion criteria were assessed using a checklist of dichotomous items.

We chose a sample of 50 individuals which is almost double the number used in a previous EEG study combining cortical connectivity with AAI^20^ and which showed acceptable power even to detect large size differences. Given the low prevalence (i.e., approximately 17%) of U/D individuals in student samples^13^ and the time and expense associated with administering the AAI^51–53^, 146 participants were pre-screened for quality of parenting using the Measure of Parenting Style (MOPS^54^), a self-report questionnaire assessing mother’ and father’ perceived parental styles (i.e., indifference, over-control, and abuse) during their first 16 years of life. According to the empirical data showing the association between dysfunctional parenting and unresolved/disorganized attachment^55^, in order to identify two groups with a similar ratio of U/D and O/R participants, of the 146 individuals pre-screened, we enrolled for the experimental session (i.e., EEG recordings and AAI) a convenience sample of 50 participants (36 females, mean age = 22.62 ± 2.41 years) scoring in the lowest (N = 25) and highest (N = 25) portions of the MOPS scores distribution. Specifically, the 146 participants were divided into two groups based on the median scores of the MOPS maternal and paternal scales (6 and 5, respectively). Sixty-three participants (i.e., 43.2%) who scored both ≥ 6 and ≥ 5 were classified as having highly dysfunctional parenting. In contrast, eighty-three (i.e., 56.8%) individuals were classified as having low dysfunctional parenting. Finally, participants from the lowest (i.e., N = 25) and highest portions (i.e., N = 25) of the MOPS score distribution were randomly selected. These participants are the same involved in our previous EEG study^56^. All participants provided their written informed consent. The study was conducted according to the Helsinki declaration standards and the research project was approved by the European University’s ethics review board.

### Experimental procedure

The experiment was conducted on two different days. On the first day, after providing written informed consent, participants completed an inclusion/exclusion criteria checklist (already used in^57^) and a battery of self-report questionnaires in their Italian version, including: the Brief Symptom Inventory (BSI^58^), the Trait-version of the State-Trait Anxiety Inventory (STAI-T^59^), and the Beck Depression Inventory-II (BDI-II^60^). On the second day of the experimental procedure, each participant underwent the AAI^61^. For each subject, two eye-closed resting-state (RS) EEG recordings have been performed prior and after AAI (i.e., pre-AAI and post-AAI). The whole experimental procedure was carried out in the *Cognitive and Clinical Psychology Laboratory* of the European University of Rome.

The BSI^62^ is a 53-item inventory (Likert scale ranging from 0 = *not at all* to 4 = *extremely*) assessing general psychopathological symptoms experienced in the last 7 days. This inventory provides 9 symptomatologic dimensions and a general measure of psychological distress (i.e., Global Severity Index, GSI). The Cronbach’s alpha in the current sample was 0.94 for the GSI.

The STAI-T^63^ is a widely used self-report inventory to evaluate trait-anxiety composed of 20 items rated on a 4-point Likert scale (1 = almost never; 4 = almost always), with total score ranging 20–80 (higher scores reflect higher levels of trait anxiety). The Cronbach’s alpha in the current sample was 0.92 for the STAI-T total score.

The BDI-II^64^ is a self-report assessing depressive symptoms in the 2 weeks prior to administration. It is composed by 21 items on which responses are rated on a 4-point Likert scale which ranges from 0 to 3 (0 = not at all; 3 = extremely), such that higher scores reflect higher depressive symptoms (total score range = 0–63). In the present sample, the Cronbach’s alpha for the total score was 0.93.

### Adult Attachment Interview (AAI)

The AAI^61^ is a semi-structured interview which requires the subject to retrieve memories of attachment experiences occurred during childhood (i.e., emotional and relational) triggering the activation of attachment system^10,41,61^. This clinical interview has been developed to evaluate the attachment related current state of mind respect to attachment experiences with caregivers^9^. Previous rigorous works (i.e., meta-analyses, psychometric studies) on the AAI demonstrated its good psychometric properties (i.e., stability, predictive and discriminant validity) in both non-clinical and clinical samples^65,66^. Accordingly, in the current study, the AAI has been used both for classifying individual’s attachment-related state of mind in terms of U/D vs. O/R, and to trigger the activation of the attachment behavioral system. The AAI was administered by a trained clinical psychologist in a quiet and comfortable room, and the average time for each interview was approximately 1 h and 30 min.

In the current study, AAI transcripts were coded by means of the Main et al.^67^ coding system by an experimenter certified (R.B.A) as reliable by Mary Main and Erik Hesse, University of Berkeley, California. As in previous works (e.g.^68^), 20% of the AAIs (N = 10) were double-coded by another certified AAI coder (L.P.) who was also trained and certified as reliable by Mary Main and Erik Hesse. Inter-rater agreement for the two-way analysis (U/D vs. O/R) was 100%, kappa = 1.00; inter-rater agreement for the four-way analysis (Free/Autonomous, Dismissing, Entangled/Preoccupied, Unresolved and/or Cannot Classify) was 90%, kappa = 0.80. All coders were blind to all other measures and participant information. Disagreements between coders for the two-way analysis were resolved through conferencing until consensus was reached.

### EEG recordings and processing

The EEG data, acquisition procedure, and processing methods used in the current study are described in Adenzato et al.^56^. For each participant, eye-closed RS EEG recordings have been performed before and immediately after (i.e., 8–10 min for each person who takes part to the experiment) the interview, corresponding to pre-AAI and post-AAI condition, respectively. RS EEG was performed in University EEG Lab allowing rest condition (i.e., comfortable armchair, semi-darkened and isolated from noise room). Considering the possible effects of alcohol and/or caffeine on EEG traces, participants were asked not to drinking alcohol or caffeine before EEG recording session (i.e., 4/6 h before).

EEG recordings were performed with 31 standard scalp electrodes according to the 10–20 system [for detailed information about recording sites, reference electrodes, impedances, sampling frequency, band-pass filters see 56]. Artifact rejection procedure was described in detail in a previous work of our team^57^. For each participant and for each condition (i.e., before and after AAI), we consider at least 180 s of artifact-free EEG trace even if not consecutive. The Micromed System Plus digital EEGraph (Micromed© S.p.A., Mogliano Veneto, TV, Italy) was used for all EEG recordings.

### Functional connectivity and network construction

In the current study, the Fast Fourier Transform (FFT) was computed by means of the exact Low-Resolution Electromagnetic Tomography (eLORETA) software for frequency decomposition of the EEG trace considering the following frequency bands: delta (0.5–4 Hz); theta (4.5–7.5 Hz); alpha (8–12.5 Hz); beta (13–30 Hz); gamma (30.5–60 Hz). EEG connectivity analysis was performed using the eLORETA software, a validated tool for localizing brain electric activity based on multichannel surface EEG recordings^69^, which is characterized by satisfactory localization agreement with different multi-modal imaging techniques (e.g.^70–72^). The regularization parameter for eLORETA was α ≥ 0.

**Figure 1:**
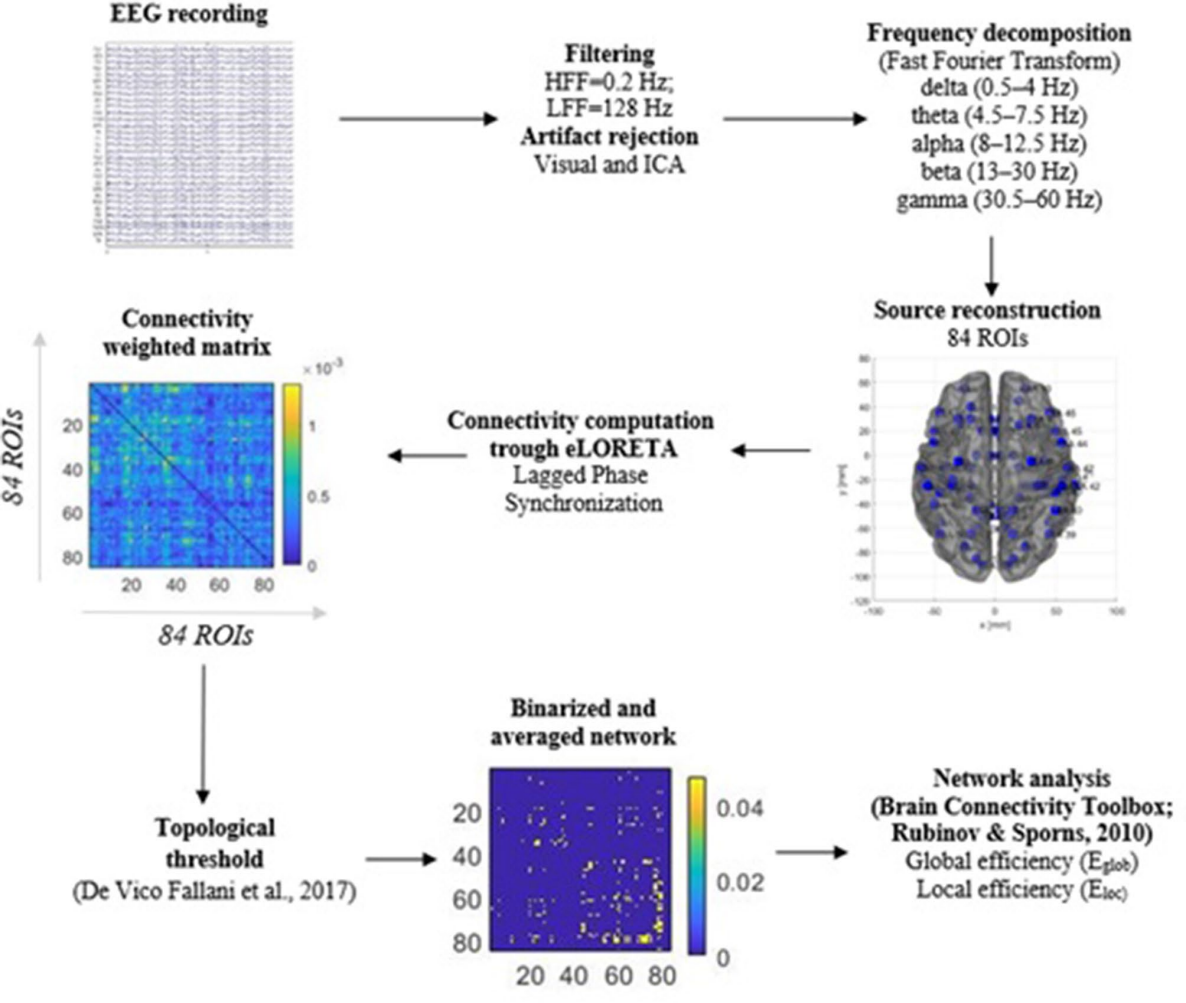
Overview of EEG recording, processing, and network analysis procedure.

According to previous studies^73–75^, by means of eLORETA and according to the Montreal Neurological Institute (MNI) atlas^76^, we computed connectivity across all 84 Regions of Interest (ROIs), 42 for each hemisphere, in order to have a complete view of the brain connectivity^73^. Specifically, we chose the “ROI-maker#2 method” available in the eLORETA software selecting 42 distinct Brodmann areas (BAs) for the left and right hemispheres. As recommended^77,78^, the “single nearest voxel” option (i.e., each ROI consisted of a single voxel, the one that is closest to the center of mass of the ROI) was chosen and the neuroanatomical MNI coordinates were computed by the eLORETA software (the table of all considered ROIs with the relative coordinates is reported in Supplementary Table 1). Then we computed the *lagged phase synchronization* (LPS) to obtain connectivity matrices. The LPS is a measure of “*similarity of two time series by means of the phases of the analyzed signal*”^79^ and its values range 0–1, respectively for no synchronization and maximum one. Connectivity matrices were used for network construction: an 84-by-84 functional network was constructed for each frequency band for each subject. More in detail, we imported connectivity weighted and undirected matrices for each subject and for each frequency band into MATLAB. These matrices reflected connectivity among 84 ROIs which represented the nodes of the network and the edges of the network were weighted by LPS connectivity. Figure 1 shows an overview of the EEG recording, processing, and network analysis procedure.

### Network analysis

After network construction, we used a topological threshold, namely “ECOfilter”^80^ in order to obtain binarized matrices on which we computed graph theoretical metrics. Specifically, as principal analyses, we computed (i) E_glob_ as a measure of functional integration^32^, (ii) E_loc_ and (iii) modularity (Q) as measures of network functional segregation^32,36^. In addition, as exploratory analyses, we computed (iv) assortativity as a measure of resilience^32,49^, and (v) betweenness centrality (BC) as a measure of node importance^32,50^.

E_glob_ estimates the efficiency of distant information exchanging in the network. It is inversely correlated with the topological distance between nodes and is described as the numerical average of the inverse shortest path length between all pairs of nodes in the network^28,32^. Therefore, higher E_glob_ values reflect higher capability of parallel information exchanging and higher integration of processes^81^.

E_loc_ is a measure related to the clustering coefficient and described as the E_glob_ calculated on the neighborhood of nodes^28,32^. In the context of complex functional networks such as the brain, E_loc_ is considered a measure of functional segregation^32^. In the current study, the local efficiency of the entire network (E_loc_) was computed as the average of the local efficiency over all nodes in the network.

Modularity (Q) is a metric that indicates the extent to which it is possible to subdivide the network into non-overlapping and defined groups of nodes^29–31^, and it is considered a global measure of network segregation^32^.

Assortativity is a measure of the extent to which network nodes with many connections are more likely to be linked to nodes with low (disassortative networks) or many (assortative networks) connections^49^. Accordingly, highly assortative networks seems to be more resilient, while disassortative ones (e.g., biological ones) less, thus being more vulnerable^49^. In an assortative network “*the nodes (…) many connections tend to be connected to other nodes with many connections*” [49; p. 208,701–1]. Thus, the assortativity coefficient (A_c_)^49^ can be seen as “*a correlation coefficient between the degrees of all nodes on two opposite ends of a link*” (^32^; p. 1065) with a positive A_c_ indicating a more resilient network and a negative one indicating a more vulnerable system^32,49^.

Lastly, the BC is a network metric characterizing the importance of nodes in bridging different parts of the network^50^. Mathematically, it is “*the fraction of all shortest paths in the network that pass through a given node*” [32; p. 1064]. Accordingly, a high BC is often associated to nodes with a function in connecting distant network’s elements^32^. Specifically, in the current work, this metric has been computed as the average of all the nodal BC values. Thus, higher BC values indicate that, on average, more nodes in the network are part of more shortest paths. Conversely, lower BC values indicate that lesser nodes in the network are part of a lower number of shortest paths.

All network metrics have been computed by using the Brain Connectivity Toolbox (http://www.brain-connectivity-toolbox.net)^32^ for MATLAB (http://www.mathworks.com).

### Statistical analyses

According to a previous study^19^ two groups were defined on the basis of their state of mind organization in relation to attachment: individuals with organized state of mind in relation to attachment (O/R), and individuals with disorganized organized state of mind in relation to attachment (U/D). Differences between groups for clinical and sociodemographic variables were investigated using independent *t* tests and chi-squared (χ^2^) tests for dichotomous and dimensional measures, respectively.

To investigate the effect of retrieval of attachment memories on brain network metrics across frequency bands in U/D vs O/R participants, we used analysis of covariance (ANCOVA). Specifically, the brain network metrics for each frequency band at T1 (i.e., post AAI) were considered as dependent variables, with group (i.e., O/R vs U/D participants) as a between-subject factor. Network metrics for each frequency band at T0 (i.e., pre AAI) were included as covariates to control for baseline EEG values (see for example^82–84^). Due to the different network properties (i.e., small-worldness) showed by males and females in EEG RS networks^85^, we also added gender as a further covariate. Finally, in the case of differences in age and measures of general psychological distress (GSI), depression, or anxiety, we add these variables as additional covariates.

E_glob_, E_loc_, and Q ANCOVAs were performed separately, applying a formal Bonferroni correction for each family of comparisons by dividing the limit of significance by the number of comparisons (i.e., five frequency bands: delta, theta, alpha, beta and gamma). Thus, the threshold level for significance was *p* = .05/5 = .01. To determine the effect size, Cohen's *d*_*ppc2*_ values were calculated (^86^; equation number 8). Similarly, also for A_c_ and BC the same statistical analyses have been performed. All statistical analyses were performed using SPSS (version 18.0).

## Results

AAI coding shows that, of the total sample (i.e., n = 50), n = 29 individuals were classified as Organized/Resolved (O/R = 58%; 22 females) and n = 21 individuals were classified as having Unresolved/Disorganized (U/D = 42%; 14 females) state of mind in relation to attachment. Indicators of state-of-mind disorganization in relation to attachment or unresolved state of mind were associated with experiences of abuse and/or loss in relation to primary attachment figures. In the U/D group, n = 19 interviews were classified primarily as Unresolved (U), and n = 2 were classified as Cannot Classify (CC). In the O/R group, n = 8 were classified primarily as Free/Autonomous, n = 16 as Dismissing, and n = 5 as Entangled/Preoccupied. Sociodemographic and clinical data for both groups are shown in Table 1. The groups did not differ with respect to gender and education level, but they did differ with respect to age (*p* = .022). The groups did not differ with respect to other clinical variables such as general psychological distress (GSI), depression (BDI) and trait anxiety (STAI-T). As expected, the U/D group had a higher MOPS total score compared to the O/R group (*p* = .009).

**Table 1:** Clinical and sociodemographic features of groups.

	O/R (N = 29)	U/D (N = 21)	Test	*p*
Females—N (%)	22 (75.86%)	14 (66.66%)	χ^2^ = .511	.475
Age—M ± SD	21.90 ± 1.63	23.62 ± 2.94	t_28.90_ = −2.43	**.022**
Education (years)—M ± SD	15.24 ± 1.88	16.00 ± 1.52	t_48_ = −1.52	.135
MOPS—M ± SD	13.72 ± 12.63	28.48 ± 21.49	t_29.92_ = −3.05	**.009**
BDI-II—M ± SD	11.41 ± 9.85	16.19 ± 12.42	t_48_ = −1.52	.136
STAI-T—M ± SD	46.90 ± 12.08	48.00 ± 15.17	t_48_ = −.29	.776
GSI—M ± SD	.68 ± .75	.72 ± .63	t_48_ = −.19	.851

*M* mean, *SD* standard deviation, *F* females, *O/R* organized state of mind in relation to attachment, *U/D* unresolved/disorganized state of mind in relation to attachment, *MOPS* measure of parenting style, *BDI-II* Beck Depression Inventory-II, *STAI-T* Trait scale of State and Trait Anxiety Inventory, *GSI* Global Severity Index.

Significant values in bold.

**Figure 2:**
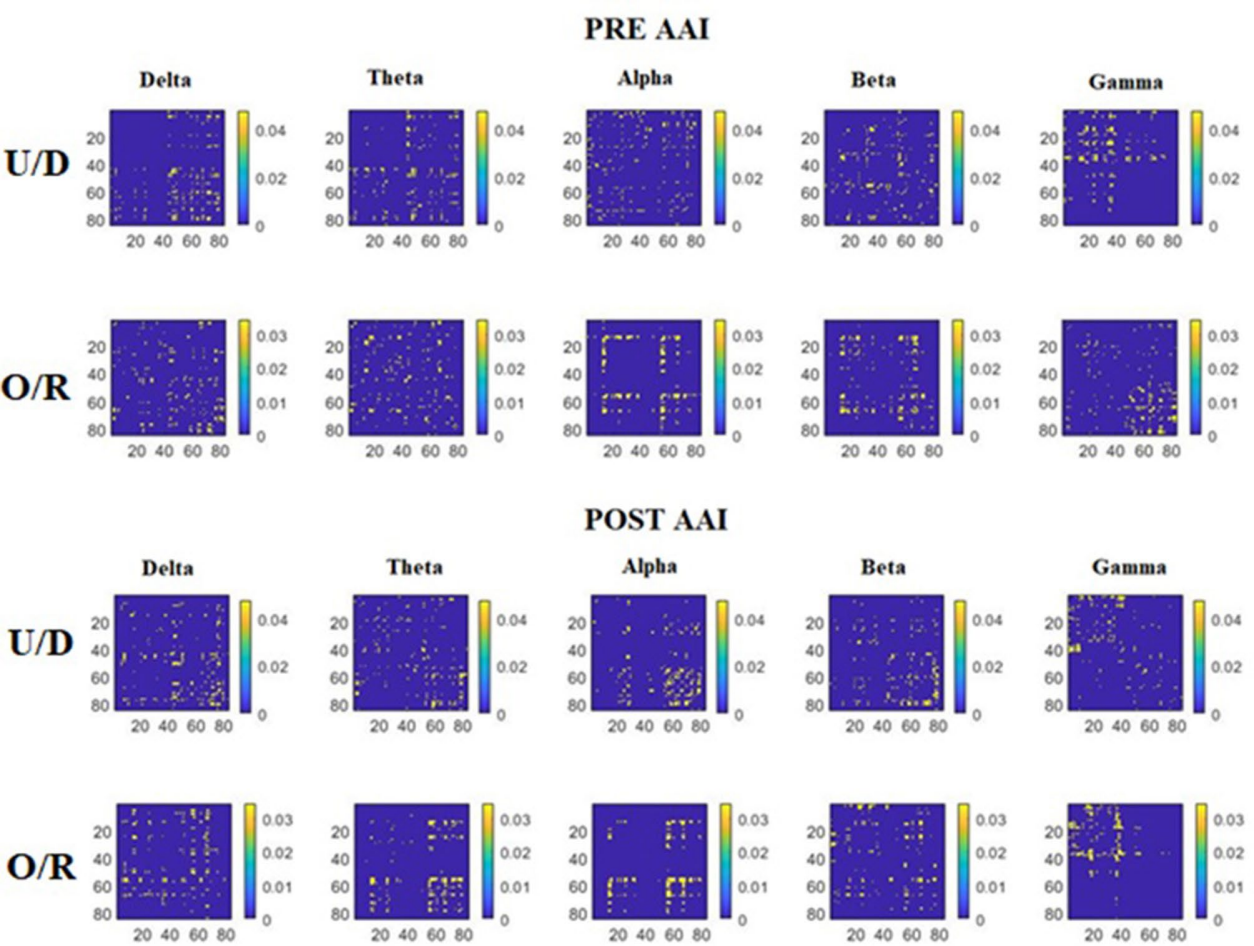
Averaged network matrices for all standard frequency bands (i.e., delta, theta, alpha, beta and gamma) for both conditions (i.e., pre AAI and post AAI), for both groups (i.e., *U/D*  unresolved/disorganized state of mind in relation to attachment group, *O/R* organized/resolved state of mind in relation to attachment group). Points on matrices axes represent ROIs going from 1 to 84.

Regarding the functional connectivity results, Fig. 2 shows the averaged network matrices in the two conditions considered (i.e., pre-AAI and post-AAI) for both groups (i.e., O/R and U/D). The connectivity weighted matrices for both groups, conditions, and all frequency bands are shown in Supplementary Fig. 1. As regarding brain network measures, ANCOVA analysis (Table 2) shows that groups significantly differ in E_glob_ metric post-AAI in beta frequency band [O/R group = .152 ± .040 (M ± SD); U/D group = .125 ± .031 (M ± SD); F_T1_(1;49) = 7.60; *p* = .008]. ANCOVA analysis showed no significant differences between groups for either E_loc_ (Table 3) and the Q metric (Table 4). We also did not find statistically significant differences between groups for the A_c_ and BC metrics. Only a tendency near to significance has emerged for the BC metric in the beta frequency band. Full data on A_c_ and BC results are presented in Supplementary Tables 2 and 3.

**Table 2:** ANCOVAs results for global efficiency (E_glob_).

Variable	Time	O/R (N = 29) M ± SD	U/D (N = 21) M ± SD	Test statistics	Cohen’s *d*_*ppc2*_
E_glob_—delta	T0	.167 ± .043	.164 ± .032	F_T1_(1;49) = 0.11; *p* = .740	.025
	T1	.158 ± .037	.154 ± .045		
E_glob_—theta	T0	.161 ± .039	.161 ± .038	F_T1_(1;49) = 0.07; *p* = .797	.026*
	T1	.163 ± .031	.164 ± .040		
E_glob_—alpha	T0	.140 ± .027	.131 ± .024	F_T1_(1;49) = 4.13 *p* = .048	.382
	T1	.142 ± .033	.123 ± .034		
E_glo_—beta	T0	.157 ± .035	**.**153 ± .033	**F**_**T1**_**(1;49) = 7.60; *p* = .008**	**.662**
	**T1**	**.152 ± .040**	**.125 ± .031**		
E_glob_—gamma	T0	.151 ± .034	.141 ± .036	F_T1_(1;49) = 0.12; *p* = .728	.452*
	T1	.147 ± .042	.153 ± .035		

Mean and standard deviation values for T1 are not adjusted for covariates (i.e., gender, age, and baseline EEG brain network metrics).

Significant tests are reported in bold.

*M* mean, *SD* standard deviation, *E*_*glob*_ global efficiency, *O/R* organized/resolved group, *U/D* unresolved/disorganized group, *T0* pre Adult Attachment Interview, *T1* post Adult Attachment Interview.

*Absolute value.

**Table 3:** ANCOVAs results for local efficiency (E_loc_).

Variable	Time	O/R (N = 29) M ± SD	U/D (N = 21) M ± SD	Test statistics	Cohen’s *d*_*ppc2*_
E_loc_—delta	T0	.095 ± .062	.099 ± .058	F_T1_(1;49) = .37; *p* = .548	.114*
	T1	.104 ± .050	.115 ± .057		
E_loc_—theta	T0	.098 ± .053	.100 ± .055	F_T1_(1;49) = 1.25; *p* = .269	.512
	T1	.136 ± .047	.110 ± .056		
E_loc_—alpha	T0	.160 ± .071	.159 ± .062	F_T1_(1;49) = .461; *p* = .501	.175*
	T1	.161 ± .073	.172 ± .076		
E_loc_—beta	T0	.148 ± .043	.143 ± .056	F_T1_(1;49) = 1.854; *p* = .180	.524*
	T1	.137 ± .067	.158 ± .047		
E_loc_—gamma	T0	.150 ± .066	.160 ± .067	F_T1_(1;49) = .01; *p* = .941	.163
	T1	.142 ± .053	.141 ± .065		

Mean and standard deviation values for T1 are not adjusted for covariates (i.e., gender, age, and baseline EEG brain network metrics).

*M*  mean, *SD* standard deviation, *E*_*loc*_ local efficiency, *O/R* organized/resolved group, *U/D* unresolved/disorganized group, *T0* pre Adult Attachment Interview, *T1* post Adult Attachment Interview.

*Absolute value.

**Table 4:** ANCOVA's results for modularity (Q).

Variable	Time	O/R (N = 29) M ± SD	U/D (N = 21) M ± SD	Test statistics	Cohen’s *d*_*ppc2*_
Q—delta	T0	.390 ± .115	.382 ± .111	F_T1_(1;49) = .013; *p* = .911	.130*
	T1	.362 ± .120	.369 ± .132		
Q—theta	T0	.392 ± .106	.392 ± .114	F_T1_(1;49) = .053; *p* = .819	.063
	T1	.407 ± .094	.400 ± .113		
Q—alpha	T0	.324 ± .091	.287 ± .097	F_T1_(1;49) = 1.706; *p* = .198	.116
	T1	.307 ± .083	.259 ± .096		
Q—beta	T0	.394 ± .071	.378 ± .105	F_T1_(1;49) = 2.756; *p* = .104	.329
	T1	.376 ± .104	.331 ± .105		
Q—gamma	T0	.369 ± .109	.333 ± .123	F_T1_(1;49) = 1.071; *p* = .306	.659*
	T1	.357 ± .119	.398 ± .106		

Mean and standard deviation values for T1 are not adjusted for covariates (i.e., gender, age, and baseline EEG brain network metrics).

*M* mean, *SD* standard deviation, *Q* maximized modularity, *O/R* organized/resolved group, *U/D* unresolved/disorganized group, *T0* pre Adult Attachment Interview, *T1* post Adult Attachment Interview.

*Absolute value.

## Discussion

The aim of the current study was to examine changes in network metrics across frequency bands following exposure to AAI in individuals with organized and disorganized attachment-related state of mind. To our knowledge, this is the first study to examine attachment-related brain network metrics following activation of affective and cognitive processes associated with attachment.

Two main results have emerged. Partly in agreement with our hypothesis, the results show that, after exposure to AAI, compared with individuals with O/R state of mind, the individuals with U/D showed reduced global efficiency in the beta frequency band. There were no statistically significant differences in the other frequency bands examined. Contrary to expectations, there were no significant differences in local efficiency and modularity when comparing the groups after AAI. Similarly, no statistically significant differences emerged for the metrics of assortativity and betweenness centrality, suggesting that activation of attachment memories is more likely to affect cortical connectivity for the integrative network metric than for the metrics of segregation, resilience, and centrality. These results appear particularly interesting given the absence of differences in clinical variables (i.e., BDI, STAI-T, and GSI scores). In other words, our results suggest that U/D state of mind in absence of psychiatric diagnosis or psychopathological conditions could affect integration but not segregation and alter optimal mental functioning based on the balance between segregation and integration of information^33–35^.

In agreement with previous studies^20,56^, these results suggest that AAI is a very demanding task that requires highly integrated mental activity in terms of cognitive and affective processes and an increase in global efficiency that may be associated with increased widespread functional connectivity in healthy individuals. Since global efficiency is considered a measure of network integration^27^, our results may shed light on pathogenic functional network processes related to activation of attachment memories in individuals with U/D state of mind without psychiatric disorder.

It has been reported in the literature that high-frequency oscillations (i.e., beta and gamma oscillations) and, in particular, dynamic, large-scale brain networks operating at these frequencies appear to be involved in higher-order cognitive processes, including sensorimotor integration, consciousness, attention, memory, and affective processes^42–46^. Indeed, it is well known that high globally efficient information exchange across all the network and functional connectivity would promote highly integrated mental processes such as emotional awareness^87^ and emotion regulation^44,88^, executive cognitive functions^81^, social cognition and mentalization^89^ and self-related mental processes such as autobiographical memory, self-consciousness and its properties of self-coherence, self‐continuity, self-embodiment, and sense of agency^88,90^.

According to this literature, our finding seems to suggest that the decrease in cortical global efficiency observed in the U/D group may reflect state-dependent impairments in higher-order affective, cognitive, and metacognitive processes triggered by traumatic attachment memories of AAI and affecting mentalization, autobiographical memory, and emotion regulation. Consistently, U/D state of mind is associated with high-level alterations in integrative mental functioning, leading to impairments in cognitive, meta-cognitive and emotional regulation^11,20,56,91^. Indeed, individuals with U/D attachment-related state of mind show signs of impaired mental integration when discussing potentially traumatic experiences (e.g., transient alterations in consciousness)^9,92^.

From a clinical point of view, several authors hypothesized that the repeated threatening and contradictory interactions of attachment disorganization could lead to a simultaneous and competitive activation of the attachment and defensive systems at the same moment towards the same person^10,16,93^, resulting in an intersubjective process that hamper the integrated and organized development of mental functions^15,94^. Therefore, it has been suggested that, in individuals with U/D states of mind, activation of attachment memories may act as a traumatic trigger for disintegrative processes that impair optimal functioning of higher-order mental functions in ways that are not different from other trauma-related pathogenic processes involving executive and regulatory functions, self-related processes, social cognition, and mentalization^11,20,56,90,95^.

The present results seem to be in agreement with previous studies reporting altered integration between cortical and cortical-subcortical structures in clinical samples^1,20,23^. However, there are some differences worth investigating.

For example, a previous EEG study on functional connectivity^20^ reported that dissociative patients had lower functional connectivity among widespread cortical regions in high frequency bands after exposure to AAI, compared to controls. This suggests that this pattern may reflect poor integrative mental processes required during retrieval of attachment-related memories, which are known to be impaired in these patients, but also more generally in individuals with a disorganized state of mind in relation to attachment^9,10^. Nevertheless, in our study we did not observe changes in segregation metrics, that we expected to be indicative of dissociation. It could be argued that our sample in the present study did not show clinical symptoms of dissociation and that the possible relationship between segregation changes and dissociation should be investigated in clinical samples with compartmentalization symptoms^96^. Indeed, Liotti^97^ hypothesized that the threatening and paradoxical intersubjective experience of disorganized attachment may constitute only a predisposition to later dissociation regarded as compartmentalization of self-experiences and that other subsequent and long-lasting traumatic experiences after early disorganized attachment are necessary for clinically relevant dissociative symptoms to develop. This hypothesis has been supported by controlled longitudinal studies^98,99^. We, therefore, argue that in our non-clinical sample disorganized attachment-related memories temporarily impaired global integrative capacity without affecting the more stable segregation functioning that likely plays a role in dissociative disorders^11^.

Although potentially interesting, our study has some limitations related to EEG, such as the small number of electrodes considered and the intrinsic limit of EEG, which is known to have poor spatial resolution. Future studies should overcome these limitations and investigate integrative/disintegrative processes and, more generally, the topology of functional brain networks related to attachment by considering a broader range of network metrics, including nodal ones to gain a more comprehensive insight into the functioning of large-scale networks in individuals with different attachment-related states of mind after activation of the attachment system. Moreover, future studies should either replicate or discard these data in larger and clinical (e.g., patients with borderline personality disorder) samples. In conclusion, even if there are some aspects limiting the generalizability of these findings, to our knowledge, this is the first study exploring network metrics such as those of efficiency following exposure to AAI in individuals with disorganized and organized state of mind in relation to attachment. Our results show that, compared to individuals with O/R attachment-related state of mind, those with U/D have lower global efficiency after exposure to AAI suggesting poor network integration. These data may help to better understand the neurophysiological patterns underlying the disintegrative effects of retrieving traumatic attachment memories in individuals with disorganized state of mind in relation to attachment.

## Supplementary Information

Supplementary Figure 1.

Supplementary Table 1.

Supplementary Table 2.

Supplementary Table 3.

## Data Availability

The data that support the findings of this study are available from the first author upon request.
