# Supplementary material for: Decreased brain network global efficiency after attachment memories retrieval in individuals with unresolved/disorganized attachment-related state of mind
Source: Sci Rep. 2022 Mar 18;12:4725. doi: 10.1038/s41598-022-08685-0 (PMC8933467; doi:10.1038/s41598-022-08685-0)
Supplement: Supplementary file 1 — Supplementary Figure 1. [file 41598_2022_8685_MOESM1_ESM.docx]

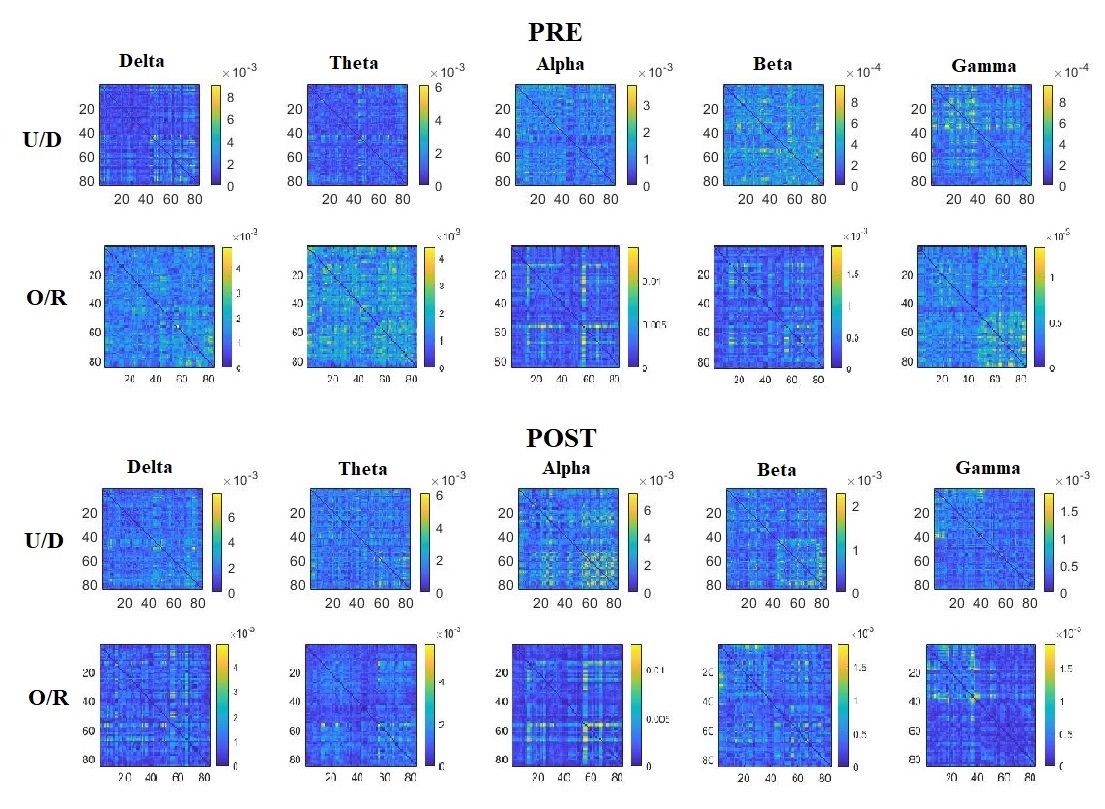


**Supplementary Figure 1**. Weighted network matrices for all standard frequency bands (i.e., delta, theta, alpha, beta and gamma) for both conditions (i.e., pre AAI and post AAI), for both groups (i.e., U/D= unresolved/disorganized state of mind in relationship to attachment group, O/R= organized/resolved state of mind in relationship to attachment group). Points on matrices axes represents ROIs going from 1 to 84.
