# Supplementary material for: Decreased brain network global efficiency after attachment memories retrieval in individuals with unresolved/disorganized attachment-related state of mind
Source: Sci Rep. 2022 Mar 18;12:4725. doi: 10.1038/s41598-022-08685-0 (PMC8933467; doi:10.1038/s41598-022-08685-0)
Supplement: Supplementary file 2 — Supplementary Table 1. [file 41598_2022_8685_MOESM2_ESM.docx]

**Supplementary table 1.** Regions of Interest considered in network construction.

| **ROI** | **BA** | **Z** | **X** | **Y** | **Structure** |
| --- | --- | --- | --- | --- | --- |
| **ROI 1** | 1 left | 50 | -53 | -25 | Post-central gyrus |
| **ROI 2** | 1 right | 50 | 55 | -24 | Post-central gyrus |
| **ROI 3** | 2 left | 47 | -47 | -30 | Post-central gyrus |
| **ROI 4** | 2 right | 47 | 48 | -30 | Inferior parietal lobule |
| **ROI 5** | 3 left | 53 | -37 | -27 | Pre-central gyrus |
| **ROI 6** | 3 right | 52 | 40 | -27 | Post-central gyrus |
| **ROI 7** | 4 left | 51 | -37 | -22 | Pre-central gyrus |
| **ROI 8** | 4 right | 52 | 37 | -23 | Post-central gyrus |
| **ROI 9** | 5 left | 60 | -17 | -44 | Pre-central lobule |
| **ROI 10** | 5 right | 59 | 13 | -43 | Lobulo para-centrale |
| **ROI 11** | 6 left | 53 | -27 | -3 | Middle frontal gyrus |
| **ROI 12** | 6 right | 54 | 27 | -3 | Middle frontal gyrus |
| **ROI 13** | 7 left | 50 | -17 | -63 | Precuneus |
| **ROI 14** | 7 right | 49 | 15 | -63 | Precuneus |
| **ROI 15** | 8 left | 49 | -22 | 28 | Superior frontal gyrus |
| **ROI 16** | 8 right | 49 | 20 | 29 | Superior frontal gyrus |
| **ROI 17** | 9 left | 33 | -29 | 30 | Superior frontal gyrus |
| **ROI 18** | 9 right | 33 | 28 | 32 | Middle frontal gyrus |
| **ROI 19** | 10 left | 9 | -22 | 54 | Superior frontal gyrus |
| **ROI 20** | 10 right | 9 | 22 | 54 | Superior frontal gyrus |
| **ROI 21** | 11 left | -17 | -18 | 43 | Middle frontal gyrus |
| **ROI 22** | 11 right | -17 | 19 | 43 | Superior frontal gyrus |
| **ROI 23** | 13 left | 9 | -39 | -8 | Insula |
| **ROI 24** | 13 right | 9 | 40 | -7 | Insula |
| **ROI 25** | 17 left | -1 | -12 | -90 | Lingual gyrus |
| **ROI 26** | 17 right | 0 | 12 | -90 | Lingual gyrus |
| **ROI 27** | 18 left | 1 | -17 | -85 | Lingual gyrus |
| **ROI 28** | 18 right | 2 | 14 | -85 | Lingual gyrus |
| **ROI 29** | 19 left | 9 | -28 | -76 | Cuneus |
| **ROI 30** | 19 right | 9 | 29 | -76 | Cuneus |
| **ROI 31** | 20 left | -29 | -47 | -22 | Fusiform gyrus |
| **ROI 32** | 20 right | -29 | 47 | -22 | Fusiform gyrus |
| **ROI 33** | 21 left | -15 | -57 | -18 | Middle temporal gyrus |
| **ROI 34** | 21 right | -15 | -58 | -17 | Middle temporal gyrus |
| **ROI 35** | 22 left | 5 | -56 | -25 | Superior temporal gyrus |
| **ROI 36** | 22 right | 3 | 56 | -22 | Superior temporal gyrus |
| **ROI 37** | 23 left | 24 | -6 | -40 | Posterior cingulate |
| **ROI 38** | 23 right | 24 | 4 | -43 | Posterior cingulate |
| **ROI 39** | 24 left | 36 | -8 | 2 | Cingulate gyrus |
| **ROI 40** | 24 right | 36 | 7 | 1 | Cingulate gyrus |
| **ROI 41** | 25 left | -17 | -7 | 17 | Middle frontal gyrus |
| Abbreviations: ROI = Region of Interest; BA = Brodmann Area. | | | | | |

**Table 4.** cont.

| **ROI** | **BA** | **Z** | **X** | **Y** | **Structure** |
| --- | --- | --- | --- | --- | --- |
| **ROI 42** | 25 right | -14 | 5 | 14 | Subcallosal gyrus |
| **ROI 43** | 27 left | -4 | -19 | -33 | Parahippocampal gyrus |
| **ROI 44** | 27 right | -4 | 18 | -33 | Parahippocampal gyrus |
| **ROI 45** | 28 left | -24 | -20 | -9 | Parahippocampal gyrus |
| **ROI 46** | 28 right | -23 | 21 | -9 | Parahippocampal gyrus |
| **ROI 47** | 29 left | 7 | -7 | -50 | Posterior cingulate |
| **ROI 48** | 29 right | 7 | 6 | -50 | Posterior cingulate |
| **ROI 49** | 30 left | 5 | -13 | -58 | Posterior cingulate |
| **ROI 50** | 30 right | 7 | 12 | -57 | Cuneus |
| **ROI 51** | 31 left | 32 | -11 | -50 | Precuneus |
| **ROI 52** | 31 right | 33 | 9 | -48 | Precuneus |
| **ROI 53** | 32 left | 21 | -9 | 29 | Anterior cingulate |
| **ROI 54** | 32 right | 20 | 8 | 30 | Anterior cingulate |
| **ROI 55** | 33 left | 23 | -5 | 15 | Anterior cingulate |
| **ROI 56** | 33 right | 23 | 3 | 17 | Anterior cingulate |
| **ROI 57** | 34 left | -19 | -18 | 1 | Parahippocampal gyrus |
| **ROI 58** | 34 right | -19 | 18 | 1 | Parahippocampal gyrus |
| **ROI 59** | 35 left | -20 | -22 | -25 | Parahippocampal gyrus |
| **ROI 60** | 35 right | -21 | 23 | -25 | Parahippocampal gyrus |
| **ROI 61** | 36 left | -24 | -31 | -28 | Parahippocampal gyrus |
| **ROI 62** | 36 right | -24 | 30 | -26 | Parahippocampal gyrus |
| **ROI 63** | 37 left | -14 | -46 | -54 | Fusiform gyrus |
| **ROI 64** | 37 right | -14 | 46 | -54 | Fusiform gyrus |
| **ROI 65** | 38 left | -27 | -39 | 13 | Superior temporal gyrus |
| **ROI 66** | 38 right | -28 | 39 | 13 | Superior temporal gyrus |
| **ROI 67** | 39 left | 24 | -46 | -66 | Medial temporal gyrus |
| **ROI 68** | 39 right | 24 | 46 | -65 | Medial temporal gyrus |
| **ROI 69** | 40 left | 40 | -49 | -43 | Inferior parietal lobule |
| **ROI 70** | 40 right | 41 | 50 | -43 | Inferior parietal lobule |
| **ROI 71** | 41 left | 10 | -46 | -29 | Trasversal temporal gyrus |
| **ROI 72** | 41 right | 10 | 47 | -29 | Trasversal temporal gyrus |
| **ROI 73** | 42 left | 12 | -62 | -23 | Superior temporal gyrus |
| **ROI 74** | 42 right | 12 | 63 | -24 | Superior temporal gyrus |
| **ROI 75** | 43 left | 16 | -57 | -12 | Trasversal temporal gyrus |
| **ROI 76** | 43 right | 15 | 58 | -10 | Trasversal temporal gyrus |
| **ROI 77** | 44 left | 14 | -52 | 9 | Precentral gyrus |
| **ROI 78** | 44 right | 14 | 53 | 9 | Precentral gyrus |
| **ROI 79** | 45 left | 13 | -51 | 21 | Inferior frontal gyrus |
| **ROI 80** | 45 right | 13 | 52 | 21 | Inferior frontal gyrus |
| **ROI 81** | 46 left | 18 | -45 | 36 | Middle frontal gyrus |
| **ROI 82** | 46 right | 18 | 47 | 36 | Middle frontal gyrus |
| **ROI 83** | 47 left | -13 | -34 | 25 | Inferior frontal gyrus |
| **ROI 84** | 47 right | -13 | 34 | 25 | Inferior frontal gyrus |
| Abbreviations: ROI = Region of Interest; BA = Brodmann Area. | | | | | |
