# Supplementary material for: Decreased brain network global efficiency after attachment memories retrieval in individuals with unresolved/disorganized attachment-related state of mind
Source: Sci Rep. 2022 Mar 18;12:4725. doi: 10.1038/s41598-022-08685-0 (PMC8933467; doi:10.1038/s41598-022-08685-0)
Supplement: Supplementary file 3 — Supplementary Table 2. [file 41598_2022_8685_MOESM3_ESM.docx]

**Supplementary Table 2.** ANCOVA's results for assortativity (A_c_).

| Variable | Time | O/R (N= 29)  M±SD | U/D (N= 21) M±SD | Test statistics | Cohen’s *d_ppc2_* |
| --- | --- | --- | --- | --- | --- |
| A_c_ - delta | T0 T1 | -.249±.184 -.281±.230 | -.220±.187 -.232±.160 | F_T1_(1;49) =.590; *p*=.446 | .106* |
| A_c_ – theta | T0 T1 | -.183±.148 -.183±.117 | -.169±.175 -.158±.160 | F_T1_(1;49) =.668; *p*=.418 | .068* |
| A_c_ – alpha | T0 T1 | -.267±.180 -.324±.179 | -.321±.235 -.362±.169 | F_T1_(1;49) =.099; *p*=.754 | .077* |
| A_c_ – beta | T0 T1 | -.189±.135 -.238±.141 | -.265±.195 -.219±.154 | F_T1_(1;49) =1.160; *p*=.287 | .575* |
| A_c_ - gamma | T0 T1 | -.263±.198 -.290±.141 | -.268±.169 -.345±.190 | F_T1_(1;49) =.445; *p*=.508 | .264 |
| Abbreviations: M= mean, SD= standard deviation; A_c_=assortativity coefficient; O/R= organized/resolved group; U/D= unresolved/disorganized group, T0= pre Adult Attachment Interview; T1= post Adult Attachment Interview.  Notes: mean and standard deviation values for T1 are not adjusted for covariates (i.e., gender, age, and baseline EEG brain network metrics). *Absolute value. | | | | | |
