# Supplementary material for: Decreased brain network global efficiency after attachment memories retrieval in individuals with unresolved/disorganized attachment-related state of mind
Source: Sci Rep. 2022 Mar 18;12:4725. doi: 10.1038/s41598-022-08685-0 (PMC8933467; doi:10.1038/s41598-022-08685-0)
Supplement: Supplementary file 4 — Supplementary Table 3. [file 41598_2022_8685_MOESM4_ESM.docx]

**Supplementary Table 3.** ANCOVA's results for betweenness centrality (BC).

| Variable | Time | O/R (N= 29)  M±SD | U/D (N= 21) M±SD | Test statistics | Cohen’s *d_ppc2_* |
| --- | --- | --- | --- | --- | --- |
| BC - delta | T0 T1 | 72.078±40.570 57.798±31.671 | 63.971±30.482 60.493±39.934 | F_T1_(1;49) =.045; *p*=.834 | .29* |
| BC – theta | T0 T1 | 68.953±35.908 68.005±27.078 | 67.112±36.477 71.416±38.059 | F_T1_(1;49) =.061; *p*=.807 | .143* |
| BC – alpha | T0 T1 | 42.535±23.317 38.583±15.392 | 34.915±20.406 28.270±17.459 | F_T1_(1;49) =3.714; *p*=.060 | .12 |
| BC – beta | T0 T1 | 60.978±26.863 58.364±32.912 | 56.950±33.226 38.378±24.159 | F_T1_(1;49) = 5.816; *p*=.020 | .529 |
| BC - gamma | T0 T1 | 54.369±32.448 53.229±38.348 | 47.252±28.380 61.502±31.126 | F_T1_(1;49) =.677; *p*=.415 | .492* |
| Abbreviations: M= mean, SD= standard deviation; BC=betweenness centrality; O/R= organized/resolved group; U/D= unresolved/disorganized group, T0= pre Adult Attachment Interview; T1= post Adult Attachment Interview.  Notes: mean and standard deviation values for T1 are not adjusted for covariates (i.e., gender, age, and baseline EEG brain network metrics). *Absolute value. | | | | | |
